# Dying at home: a qualitative study of family carers’ views of support provided by GPs community staff

**DOI:** 10.3399/bjgp14X682885

**Published:** 2014-12-01

**Authors:** David Seamark, Susan Blake, Sarah G Brearley, Christine Milligan, Carol Thomas, Mary Turner, Xu Wang, Sheila Payne

**Affiliations:** Honiton Research Practice, Honiton.; Honiton Research Practice, Honiton.; International Observatory on End of Life Care, Division of Health Research, Lancaster University, Lancaster.; International Observatory on End of Life Care, Division of Health Research, Lancaster University, Lancaster.; International Observatory on End of Life Care, Division of Health Research, Lancaster University, Lancaster.; International Observatory on End of Life Care, Division of Health Research, Lancaster University, Lancaster.; Department of Psychology, Leeds Metropolitan University, Leeds.; International Observatory on End of Life Care, Division of Health Research, Lancaster University, Lancaster.

**Keywords:** community care, end of life, home palliative care, informal care givers, qualitative study

## Abstract

**Background:**

Dying at home is the preference of many patients with life-limiting illness. This is often not achieved and a key factor is the availability of willing and able family carers.

**Aim:**

To elicit family carers’ views about the community support that made death at home possible.

**Design and setting:**

Qualitative study in East Devon, North Lancashire, and Cumbria.

**Method:**

Participants were bereaved family carers who had provided care at the end of life for patients dying at home. Semi-structured interviews were conducted 6–24 months after the death.

**Results:**

Fifty-nine bereaved family carers were interviewed (54% response rate; 69% female). Two-thirds of the patients died from cancer with median time of home care being 5 months and for non-cancer patients the median time for home care was 30 months. An overarching theme was of continuity of care that divided into personal, organisational, and informational continuity. Large numbers and changes in care staff diluted personal continuity and failure of the GPs to visit was viewed negatively. Family carers had low expectations of informational continuity, finding information often did not transfer between secondary and primary care and other care agencies. Organisational continuity when present provided comfort and reassurance, and a sense of control.

**Conclusion:**

The requirement for continuity in delivering complex end-of-life care has long been acknowledged. Family carers in this study suggested that minimising the number of carers involved in care, increasing or ensuring personal continuity, and maximising the informational and organisational aspects of care could lead to a more positive experience.

## INTRODUCTION

Dying at home is viewed as important by patients with a reported preference of 50–60%.^1^ A recent Cochrane Review^2^ demonstrated that for patients who wish to die at home the provision of home palliative care more than doubles the chances of dying at home. However, deaths at home in the UK only account for around 20% of deaths, though there are marked regional variations.^3^ The UK Department of Health *End of Life Care Strategy* puts emphasis on enabling patients to make choices about place of death and increasing home death rates.^4^ With 83.4% of all deaths in the UK occurring in those aged >65 years there is evidence that older people can be viewed as the ‘disadvantaged dying’, with less access to health and social care than younger people.^5^ It is estimated that there are approximately half a million family carers in the UK providing care in the context of an end-of-life phase.^6^ This phase can be lengthy, with one study showing that 40% of first-degree relatives provided care for longer than 12 months.^7^ Achieving the end-of-life policy aims relies heavily on the assumption that there are family carers willing and able to provide care for the dying person.^8^,^9^ A major reason for hospital admission is a breakdown in family care,^10^ and while this central role for carers is increasingly recognised there are gaps in our knowledge about how to provide appropriate support for carers during the final phase of life.^11^

The ‘Unpacking the Home’ study was designed with the primary aim of gaining an in-depth understanding of ‘home’ and the issues faced by family members caring for a dying older person at home. A fuller description of the study and the protocol is available.^12^ One specific objective of the study was to elicit family carers’ views about the practical and other types of support that made death at home possible, and to obtain views of deficits or gaps in support. Transforming a home to a site of care for a dying person can lead to tensions. For example, there are changes to the house for a family carer and patient to contend with and also increased interactions with nurses and formal care workers (care assistants), and with out-of-hours staff. The focus of this study was to examine the family carers’ perspective of the interactions with formal care workers and primary care staff in a series of home deaths in two regions of England.

## METHOD

The 2-year qualitative study involved a multidisciplinary team combining skills from primary care, nursing, health psychology, sociology, and medical geography. It employed an inductive approach informed by the principles of grounded-theory methods and using maximum-variety sampling.^13^,^14^

**How this fits in**

The majority of patients with a life-threatening illness who express an opinion wish to die at home. Achieving the choice of a home death relies heavily on the assumption that there are family carers willing and able to provide care for the dying person. This study shows that continuity of community care provided by GPs, nursing staff, or formal carers is valued highly by family carers. In an age where personal continuity is hard to guarantee, informational and organisational continuity need to be maximised to ensure the support that family carers require.

Two study regions with a high proportion of older residents and variation in socioeconomic status were chosen: the North West (North Lancashire and Cumbria) and the South West (East Devon) of England.

### Setting and participants

Delivery of community palliative care in England involves community nurses, GPs, and formal care workers. These are supplemented in some cases with input from specialist hospice nurses based in the community and with potential help from physiotherapists, occupational therapists, and dieticians. Participants were bereaved family carers recruited through GP practices. The research team arranged meetings with GPs or other appropriate staff members (research nurses or practice managers) in each participating practice to provide verbal and written information about the study and answer questions. Practice staff undertook database searches to identify eligible family carers and posted information packs to them. Those who were interested responded directly to the research team and interviews were arranged at the participants’ homes.

The inclusion criteria were:

- family carers of older deceased people (aged ≥50 years) from any cause of anticipated death;
- death occurring in the patient’s own home or the home of the carer;
- 2-week minimum period of care prior to death;
- any age of adult carer over 18 years; and
- participants recruited at least 6 months but not more than 24 months after the death.

### Data collection

Data were collected through in-depth semi-structured interviews with bereaved carers. An interview schedule was developed by the research team to elicit chronological narratives of care provision during the dying process, death, and early period of bereavement. Interviews were digitally audiorecorded and then transcribed verbatim. Approximately 25% were double-checked by the researchers to ensure accuracy and rigour. In addition, participants were invited to write their own accounts of their experiences of caring if they wished.

### Data analysis

Analysis consisted of cross-sectional thematic analysis whereby commonalities and differences were identified both within the individual accounts and across the two study sites. An iterative approach was used, with an initial framework of thematic categories applied to interview data, drawing on the research objectives. The process of constant comparison was undertaken so that the early stages of analysis informed subsequent data collection and emergent issues were pursued throughout the research process.

## RESULTS

Of 109 bereaved family carers approached, 59 agreed to be interviewed (36 in the South West and 23 in the North West). The majority of the deceased were male (59%) and most of the carers were female (69%). Of the 59 carers, 43 were either the wife, husband, or partner of the deceased. The median length of the interview was 42 minutes. Cancer was the recorded cause of death for 37 of the patients (63%). The median length of time care was provided at home was 5 months (range 2 weeks to 108 months) for cancer patients. For patients dying from a non-cancer cause the median length of care was 30 months (range 3–132 months). The non-cancer diagnoses included heart disease, Parkinson’s disease, dementia, chronic obstructive pulmonary disease (COPD), and multiple comorbidities. There was no significant difference between the samples of carers in the North West and South West for age, ethnicity, relationship, or occupation. Home ownership, an indicator of deprivation, was lower at 78% in the North West compared with 94% in the South West.

### Study findings

Transcripts from both study areas were compared and no obvious differences in the narratives were observed. The accounts from carers looking after patients with a cancer diagnosis or a non-cancer diagnosis were comparable. Examining the data from the perspective of interactions between patients and family carers with the primary healthcare team and formal carers revealed an overarching theme of continuities of care. This theme divided into sub-themes of personal continuity, informational continuity, and organisational continuity of care, although these were often interwoven in the participants’ accounts.

#### Personal continuity

Both positive and negative aspects of personal continuity of care were reported irrespective of whether or not care was delivered by the GPs, nurses, or formal caregivers. Family carers clearly appreciated having the same one or two formal caregivers or nurses attending to the patient. This had the effect of changing the relationship from stranger to trusted helper:

‘*We had helpers from when she had, after she had her stroke, and we were very lucky. We had one lady who stayed with us for a few years. We had the same helper for a year or two. She always came at the same time. This was really to help* [A] *getting up and washing and dressing in the morning and, although obviously I could do it and did do it on holiday and when we didn’t have a helper, it just made things so much easier for me.’*

(A28 ref1)

‘Yeah, and most of them we knew because they’d been into my mum quite a lot, and so it’s quite nice when you do know them rather than strangers, though even strangers they’re always very nice. It’s always nicer to have a bit of personal contact.’

(B03 ref1)

Negative aspects were reflected in comments relating to the changing of care staff and nursing staff:

*‘She didn’t like the personal care people very much, erm … and this would be my second criticism really, that there was somebody different virtually every day, you know, and this was quite personal* [chuckles] *and I mean I certainly didn’t like it.’*

(B14 ref1)

**Interviewer (I):** *‘So did he have different people coming in all the time then?’*

‘Yes, you do tend to yeah, we had a couple if regular ones, but otherwise … you get different ones all the time.’

(B03 ref1)

According to family carers the effect of being introduced to new staff was seen as emotionally and physically draining for the ill patient. This resulted in some cases with the care offered being declined:

*‘… and we had already organised for some helpers to come in, but I left it because I knew* [G] *would hate it. He would have hated it for the simple reason, when you are very ill, there is something you can’t cope with, having every time somebody else coming, and that would have been the case. And that really was the reason why I felt I didn’t want* [G] *to put up with this …’*

(B17 ref5)

Knowing the GP and having regular contact either in person or by telephone was seen as important in providing reassurance and the sense that the family carer had not been ‘dropped’:

*‘The GP, they were all well, I mean, even the day after, both doctors* [GP2] came and [GP1] *… and I felt very grateful for that, that by the sort of medical side you weren’t completely “dropped”. There was still that care there and that I really, I have to admit, was marvellous.’*

(B17 ref7)

*‘Well* [GP1] *was absolutely brilliant, he was always on the end of a phone and you know erm whenever we were bothered and troubled by anything and worried because we didn’t know what to do, just ring the surgery, and yeah I mean something would always happen so yeah I can’t speak highly enough of them.’*

(A09 ref1)

The vast majority of care contacts were with community nurses, hospice nurses, and formal care workers. There was a negative perception that GPs were not involved in organising care and a reluctance to visit by a GP was seen in a negative light:

**I:** *‘So who organised the Marie Curie nurses then?’*

**A13:** *‘Well I don’t know, I’m not quite sure, they appeared on the scene, I don’t know if it was the district nurse. There was a district nurse and somebody told me that the district nurse does everything and the doctor doesn’t do anything these days, everything’s sort of changed.’*

(A13 ref3)

‘Nothing from the surgery, we might have had a health visitor now and again, but nothing, not after we got organised with the carers.’

(A23 ref1)

*‘Oh* [the doctor was] *very reluctant to come, I had to nearly, I was on the phone ages with him before I could get him to come mmm …’*

(B07 ref2)

Although the practical limitations of delivering home care 24 hours a day and 7 days a week were acknowledged, some reflections from family carers suggested a way to improve personal continuity of care:

‘The only thing I would say is and it’s something that isn’t practical and would never happen but it would be wonderful if one nurse could concentrate on a case because you would have that continuity and they would notice changes and things and it would help them and probably help the family in that it isn’t a different person every night and you’re having to explain where the coffee is and what to do, but I know it isn’t practical because they have to have time off. But if it were one person, or even two, because we did have several different nurses.’

(A20 ref1)

‘We are looking here about how things could be improved, and one way it could be improved is, if somebody in her position had a personal carer, who perhaps at the initial stage might just come for half an hour a day, but could then perhaps come a bit more as required and that was one person that she got on with, you know, and accepted.’

(B14 ref2)

#### Informational continuity

Overall, family carers appeared to have low expectations regarding information transfer between different agencies involved in care. For some family carers it was a pleasant surprise to discover that care details were known by out-of-hours and help agencies, and that some staff did communicate effectively:

‘I rang the emergency doctor. It was a weekend and they said, “Oh yes we were expecting a call from you.” They had all his details there so our GP must have. And that very impressed me, I was extremely impressed, and within half an hour a doctor was at his bedside. And they said, “Oh yes, he needs morphine” and he said, “I’ll arrange it.”’

(B07 ref6)

*‘Whenever I rang to* [Town1] *I got sensible answers, I mean they said, you know, they may have said, “Right, I can’t do that immediately,* [B] *or whatever it is, is out but she’ll be back in half an hour and meanwhile I’ll try and get her on the phone.” … So there wasn’t somebody sitting there all the time but all the people I contacted were in contact … with what was going on … which was very impressive actually.’*

(B20 ref5)

‘… the system was quite incredible actually, it was very well run. You would have two people here doing something and somebody else would come in, you see, probably just passing — I don’t know how they work it — and when she found that the others were there, there would be a little confab and then one or other of them would disappear again, and it would just run like that.’

(B20 ref6)

However, there were many reported negative experiences around information and communication issues. Particularly troubling were two instances where the patient had died and this information was not passed on to the care agencies involved, resulting in emotional trauma:

*‘I didn’t know she was coming in but the next morning we were sat having breakfast and* [B] *and I were talking and I suppose basically, but she came in with a young boy, the hospice nurse with a young boy that was learning and she, she didn’t know he was dead.’*

(A06 ref1)

‘And I said you know, “He’s dead”, and she said, “He can’t be.” She said, “I should have heard” and that you know. I mean she sort of stayed for 10 minutes and then went and I never seen her since.’

(A06 ref2)

**B01:** *‘But erm strangely enough when September came round the ambulance came from the hospice to take him for a week’s respite.’*

**I:** *‘Oh really?’*

**B01:** *‘No one had told them* [B] *had died.’*

**I:** *‘Oh dear.’*

**B01:** *‘And I didn’t think I had to.’*

**I:** *‘Oh dear.’*

**B01:** *‘Anyway we get over these things.’*

**I:** *‘Did you find it distressing?’*

**B01:** *‘Mmm.’*

(B01 ref1)

The need to repeat information to new nurses and formal carers was reported frequently and was frustrating to family carers:

‘I got a little bit exasperated with community nurses, what we used to call district nurses, I think, and somebody’d want to come … big questionnaire — fair enough, to try and assess the needs. Then somebody from Marie Curie came — another questionnaire — and she went away. Then somebody from the hospice came and another one … and it went on like this.’

(B14 ref2)

There was an expectation from family carers that modern information technology would allow communication between agencies and primary and secondary care. However, this was often not the case with lack of communication between GP and consultant seen as puzzling:

*‘What puzzled us one thing was there didn’t seem much contact between the doctor and the consultant, because the consultant would say “Has your doctor given you any different pills?” and we’d say either no or once we said yes, I mean there was something he said change something and we forgot to take the name of the pill in with us so* [B] *said to the chap, “If you look on your screen it will sort of be there” and the consultant said, “Oh I tell the doctor what I’ve done but we never get anything back from the doctor so I don’t know what he does.” So that seems a bit crackers. That puzzled us and this is another silly thing, we were never quite sure who was in charge of all this business, so who was in charge of it all?’*

(A13 ref1)

Many different professional carers came and seemed to lack information about the patient or had not had training related to a patient’s specific condition:

‘And dozens of different people, and some of them left the company and then new people would come who were as green as grass and would come for so-called training, which wasn’t training. And they knew nothing about the case. They would come and I would say, “Did they explain to you what he is suffering from?” “No, they didn’t say anything.” “Well, he’s suffering from Parkinson’s disease.” “Oh, yes?” you know … didn’t mean a thing to them.’

(B21 ref3)

#### Organisational continuity

Family carers were comforted and encouraged when organisational aspects functioned well both during and out of hours:

**I:** *‘How easy did you find it to contact someone if you needed help?’*

**A04:** *‘Very well most times, I mean as I say other than the holidays, but then you had these emergency doctors no problem, and they used to come out and* [GP]*, I can’t speak highly enough of her.’*

(A04 ref1)

*‘They* [doctors and nurses] *came for me a little while, the district nursing team was wonderful … they provided advice, they helped … they sort of tried to coordinate with the carers but that was difficult because, as I say, by then we didn’t know what time the carers were going to be coming. They left advice for the carers and they comforted me when I needed it.’*

(B13 ref6)

*‘The nurses then said, “We will get in touch with the hospice”, and the hospice nurses came once or twice by day, and the district nurses and the hospice nurses always knew when, so they would then sort of come and tell me, or the day before, and they would then wash* [G]*, you know, wash him, change the bed … And I knew then they were coming to do it, because I couldn’t change the bed myself.’*

(B17 ref3)

*‘And so they all* [nurses] *reacted to it without instructions or anything, absolutely fantastic. I mean the nurse used to ring up somewhere …* [City1]*, I don’t know where it was, and ask for a piece of equipment — like a thing that, a back thing that goes like in the bed so you can sit against it, and it would be here in 2 or 3 hours.’*

(B20 ref2)

However, many carers reported negative aspects related to the organisation of care. Family carers fully committed to caregiving found responsibilities for organising care or triggering a response added to their workload:

‘But then you have to plan, you have to arrange that then from then on yourselves, which we did and that only lasted for just under a week and as I say, he died.’

(A01 ref1)

‘I found that a bit hard actually to deal with because you get a list of caring companies but they aren’t all what they say they are you know, some of them are just day care centres, some of them are not what you are looking for and it’s quite hard to, sometimes they never rang back when you asked them to, but eventually we did get a company to come and we sorted it out and they came.’

(A01 ref2)

*‘It was up to me to contact her* [the hospice nurse]*, and this is what people say, if you need any help ring, but it’s an extra thing to do, to organise your own kind of help is an extra thing to do, and in the 24 hours you don’t have much time or energy for extra things.’*

(B02 ref1)

The lack of a clear action plan, not knowing who to phone and who was responsible for aspects of care, caused stress for family carers:

‘I think at the beginning, if it’d be helpful for other people, I think if things could be explained to you where you could get help from and telephone numbers, I wasn’t given anything like that at the beginning which could have been helpful for me. I was able to cope but if somebody couldn’t. I didn’t even know for example that social services came in to help you with toileting and bathing and that sort of thing, and I only find out through the nurse when she came and I thought “Oh you’ve come just at the right time” err told me, “No that’s not my role’, that rather staggered me. And I mean considering I’ve been involved with health and I didn’t even know that.’

(B07 ref15)

‘In fact, that’s another thing: they could make clearer in a way at the beginning what’s their job and what isn’t, because I’d never had help before and I didn’t know, I really didn’t know what they were there to do in the beginning. I mean I’d cared for himself, and it was … I still wanted to do it, you know, I was … and it took me a while to realise that’s their job, you leave them alone, you know … washing him and, you know, things like that. I used to try and get him toileted before they came because I thought it’s not a nice job to ask anyone to do, and then I found out, you know, that was on their rota and they didn’t mind it at all. So it would be nice if you knew at the beginning, you know.’

(B13 ref8)

Organisation of out-of-hours care attracted many negative comments with reports of situations causing distress to patient and family carer alike. In some instances a lack of response could lead to a reluctance to contact out-of-hours services and to possible avoidance of hospital admissions:

*‘Then one Sunday in May, it always happens at the weekend, erm she was very very painful* [sic]*, doubled up, so I rang the helpline which was completely useless, somebody on the end said, “Oh yes well I’ll have to speak to a doctor and we’ll get back to you”, and all the time she’s doubled up. So I waited about half an hour and then I thought this is ridiculous so I phoned the ambulance.’*

(B04 ref1)

‘If anything goes wrong during the night, weekends, they were dreadful times because at weekends the NHS more or less closes down, and you can go and sit in A&E, somebody’ll come and see you after about half an hour and take some details, but then it’s about 4 hours wait then, and if you’re sat there in pain it’s a hell of a long time.’

(B04 ref2)

‘I have a reluctance to ring the doctor at weekend but the support you get nowadays from out-of-hours doctors doesn’t seem to be, well certainly our experience, and my daughter-in-law, it seems to be a thing that I’d avoid, is ringing the doctor at a weekend if you possibly can.’

(B03 ref2)

When organised care worked well the result was that the carers felt supported and also able to be in control of the situation themselves:

‘The family felt in control of the situation and had the total support and advice of the professionals which took away the anxieties associated with death I think and I think as I’ve already said the advantage of knowing most of the nurses, apart from the Marie Curie nurses.’

(A20 ref3)

## DISCUSSION

### Summary

The importance of providing continuity of care in a community setting has been recognised for some years^15^,^16^ and promoting relational (personal) continuity is a priority for the Royal College of General Practitioners.^17^ However, effective interventions to improve continuity of care, particularly for patients with cancer, have proved elusive.^18^ The results presented here suggest that ensuring continuity of care by community staff is seen as highly important by the family carers of patients dying at home, but such care continuity was often absent. The elements of continuity — personal, informational, and organisational — have been previously described^15^,^19^ and were illustrated by the family carer accounts. Personal continuity was seen as important whether delivered by a GP, nurses, or other formal carers, though the practical limitations of delivering such care were acknowledged. The involvement of numerous healthcare workers had a negative impact on severely ill patients, sometimes resulting in refusal of support offered.

Family carers had low expectations of informational continuity, and problems with passing information on to another party were common. In particular, failure to communicate the death of a patient to other team members was a major source of upset to family carers. The lack of good communication between primary and secondary care was a source of puzzlement, a finding also reported in another study 20 examining the transition between care settings at the end of life. Family carers expressed a need for clear advice on who does what, who to phone for help, how to obtain help in an emergency, and clarification of the differences between health and social care. These were views expressed in a recent report on supporting family carers prepared for Help the Hospices.^21^

When organisational continuity was achieved, a level of comfort and confidence was expressed by family carers, and the reverse was true when it appeared disorganised. In particular, out-of-hours organisations attracted negative comments and there could be reluctance to ask for help and for potentially inappropriate outcomes such as calling an ambulance and hospital admission. Inappropriate hospital admission for patients with palliative care needs has been linked to family carers being unable to cope and lack of community services.^10^

### Strengths and limitations

A strength of this study is that it chose to examine the perspective of carers looking after patients suffering from life-limiting illness and not just cancer. The qualitative data obtained provide a rich insight into the stresses and strains of caring, which are not necessarily detected in questionnaire-based surveys.^22^ The validity of the findings is increased by purposively sampling across two areas of England where there are known to be differences in housing and living conditions, and, potentially, in availability of community care.

The retrospective design of the study can be considered a weakness, but does allow for a period of reflection by the carers interviewed, which could be argued provides a more rounded appraisal of the positive and negative aspects of providing care up to death at home. Caution is required in interpreting some of the findings as we collected no data from GPs, community nurses, or patients.

### Comparison with exisiting literature

The need for research into supporting lay carers in end-of-life care has previously been described.^11^,^23^ This qualitative study was able to elicit detailed narrative accounts of the experience of caregiving through to the final illness, including the death of the person at home. The study adds to the existing literature, which has predominantly presented evidence from the professional perspective of GPs,^24^ community nurses,^6^ and home-hospice providers.^11^ Family carers’ satisfaction with home-based nursing and physician care for cancer patients has been reported using a longitudinal questionnaire survey.^22^ Factors related to satisfaction included service providers being easy to reach, having time to listen, and treating the patient as a person and not a disease. Quick and punctual responses were also appreciated. A Dutch study examined factors associated with death with dignity and noted the importance of clear communication with physicians involved in the care.^25^ Although not qualitative studies, these findings would find resonance with our themes of personal, informational, and organisational continuity. The issues around continuity of care have been described in studies examining the out-of-hours care provided for patients in the community suffering from cancer.^26^,^27^ With the reduction in relational continuity during out-of-hours care, it has been suggested that informational and organisational continuity supplemented by good communication may enhance the experience of patients with palliative care needs.^26^ Initiatives such as the Gold Standards Framework^28^ seek to improve organisational and communication elements of home-based palliative care, and there is evidence that well-organised home palliative care can increase the chance of dying at home and reduce symptom burden without impacting on caregiver grief.^2^ Our findings suggest that where organisational and informational continuity exist, positive outcomes in terms of carer confidence and reduced anxiety may result.

### Implications for research

This study reveals that continuity of community care, whether provided by GPs, nursing staff, or formal carers, is highly valued by family carers. The challenge is how to provide continuity across the domains of personal care, good information transfer, and sound organisation. There is a need for further research as to the best models for community care that pay attention to each aspect of continuity. The issues associated with out-of-hours provision requires particular scrutiny.
